# Genome Sequences of Mycobacterium smegmatis Phages Fefferhead and ShamWow

**DOI:** 10.1128/MRA.00973-21

**Published:** 2021-11-24

**Authors:** Valerie L. Dao, Lily Xia, Christa T. Bancroft

**Affiliations:** a Department of Biological Sciences, University of Southern California, Los Angeles, California, USA; DOE Joint Genome Institute

## Abstract

Fefferhead and ShamWow are temperate mycobacteriophages in the K6 and E clusters, respectively. The length of the Fefferhead genome is 61,366 bp, with 98 predicted genes. The ShamWow genome has a length of 75,933 bp, with 143 predicted genes, 3 of which are duplicates.

## ANNOUNCEMENT

**FIG 1:**
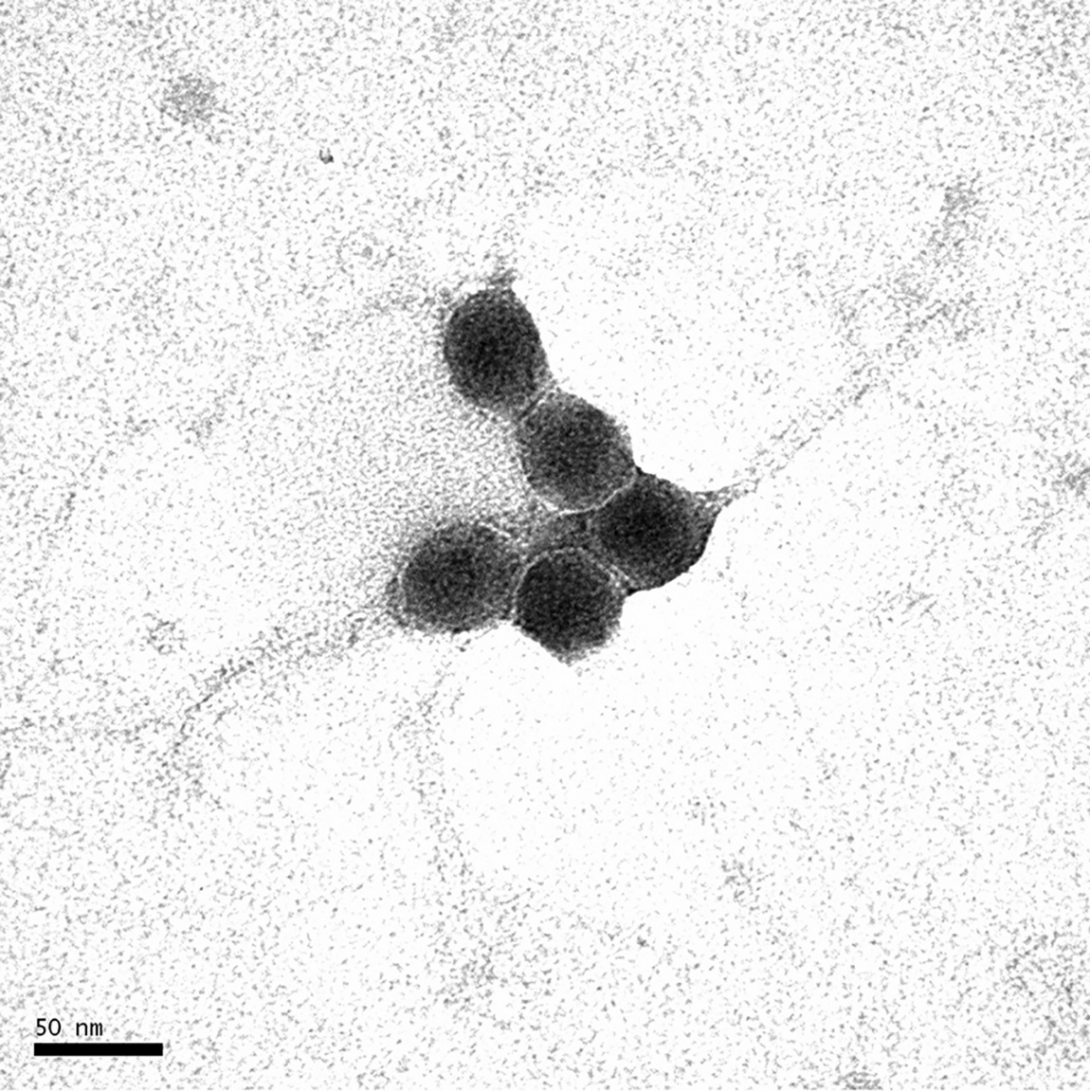
Transmission electron microscopy of bacteriophage Fefferhead.

B acteriophages are viruses that infect and replicate in bacterial hosts. Mycobacteriophages, like Fefferhead and ShamWow, are undergoing study at the University of Pittsburgh for therapeutic use against drug-resistant tuberculous and nontuberculous mycobacterial infections (1); understanding mycobacteriophage genomes is an important component of this work. Fefferhead was found by Matthew L. Petrovich in Cary, North Carolina (35.8399N, 78.8172W), and isolated in in 2014. Fefferhead was isolated using an enrichment protocol with Mycobacterium smegmatis mc^2^155 P2FF cultures grown in 7H9 medium at 32°C. After filter sterilization of the enrichment culture, Fefferhead was purified with three rounds of purification on L-agar plates containing top agar with 7H9 medium incubated at 32°C. ShamWow was found by Norhan Shamloul in Pittsburgh, Pennsylvania (40.444176N, 79.95472W), and isolated in 2012. ShamWow was isolated from a direct soil sample preparation with Mycobacterium smegmatis mc^2^155 cultures grown in 7H9 medium. ShamWow was purified with three rounds of plaque assays on 7H10 agar plates containing top agar with 7H9 medium and calcium at 37°C. Both Fefferhead and ShamWow are temperate phages, with *Siphoviridae* morphotypes identified by transmission electron microscopy and bioinformatic analysis, respectively (Fig. 1). Genomic DNA was extracted from high-titer lysates using the Promega DNA Wizard kit. At the Pittsburgh Bacteriophage Institute, sequencing libraries were prepared from genomic DNA using a NEBNext Ultra II FS kit with dual-indexed barcoding. Libraries were pooled and run on an Illumina MiSeq system, yielding 26,090 single-end 150-base reads for Fefferhead and 320,100 reads for ShamWow. Reads were assembled using Newbler v.2.9, yielding single phage contigs; the contigs were checked for completeness, accuracy, and genomic termini using Consed v.2.9 (2). The 61,366-bp genome of Fefferhead was sequenced at an average coverage depth of 58-fold, and the GC content was 67%. The 75,933-bp genome of ShamWow was sequenced at an average coverage depth of 598-fold, and the GC content was 63%.

DNA Master v.5.23.6 (3) autoannotated the genomes. Gene lengths and start sites were cross-checked across Glimmer v.3.02 (4), GeneMarkS v.2.5p (5), and Starterator v.435 (6) reports to assess start site conservation. ARAGORN (7) and tRNAscan-SE v.2.0 (8) helped predict tRNA genes. For functional assignments, Phamerator v.435 (9), HHPred v.3.2 (10), and NCBI BLAST v.2.9.0 (11) were applied. All tools were run with default parameters unless specified otherwise.

Annotations were consolidated on PECAAN v.20210219 (https://blog.kbrinsgd.org). The Fefferhead genome includes 96 annotated genes, consisting of 2 tRNA genes and 94 protein-coding genes. The ShamWow genome contains 145 annotated genes, with 2 tRNA genes and 143 protein-coding genes. The architecture of both genomes follows typical phage organization, with genes involved in structure, integration, and then DNA replication.

ShamWow genes 68, 73, and 80 are duplicates in phamily 78571, based on Phamerator v.435 (9) analysis of high amino acid similarity. This gene triplet is conserved among cluster E phages Kostya, Toto, CJW1, and Goku, exhibiting duplication in similar locations. A NCBI BLAST search returned a 99.99% identity match between the genomes of ShamWow and Toto. ShamWow and Fefferhead also share similar GC contents with other members of their respective clusters. Phamerator analysis suggests the Fefferhead gained a new gene at gene 70, compared to other cluster K6 phages.

### Data availability.

The Fefferhead genome is available in GenBank under accession number MW601222, with reads available under SRA accession numbers SRX12198767 and SRR15908340. The ShamWow genome is available in GenBank under accession number MZ274305, with reads available under SRA accession numbers SRX12345325 and SRR16058738.
